# Aspirin in the era of immunotherapy

**DOI:** 10.18632/oncotarget.20877

**Published:** 2017-09-14

**Authors:** Tsuyoshi Hamada, Marios Giannakis, Shuji Ogino

**Affiliations:** Shuji Ogino: Department of Oncologic Pathology, Dana-Farber Cancer Institute and Harvard Medical School, Program in MPE Molecular Pathological Epidemiology, Department of Pathology, Brigham and Women’s Hospital and Harvard Medical School and Department of Epidemiology, Harvard T.H. Chan School of Public Health, Boston, MA, USA

**Keywords:** clinical outcome, colorectal neoplasms, molecular pathological epidemiology, non-steroidal anti-inflammatory drugs, tumor microenvironment

Immune checkpoint inhibitors against the *PDCD1* (programmed cell death 1, PD-1)-*CD274* (*PDCD1* ligand 1, PD-L1) axis have shown unprecedented clinical benefits in the treatment of refractory neoplasms [Gentzler R, et al. Immunotherapy. 2016;8:583-600]. Recently, the U.S. Food and Drug Administration (FDA) approved the anti-*PDCD1* (PD-1) antibody pembrolizumab for treating solid tumors with high-level microsatellite instability (MSI) or mismatch repair deficiency. This approval attracted great attention as the first of an agent based on a tumor biomarker rather than the primary cancer site. High-level MSI is commonly present in colorectal carcinomas, and abundant neoantigens due to frequent frameshift mutations have been proposed as a potential explanation of the survival benefits seen with the immune checkpoint blockade in this tumor subtype [1]. However, not all MSI-high colorectal carcinomas respond to the immunotherapy. The oncogenesis of colon and rectal cancer represents a complex process that is influenced by lifestyle factors, the microbiome, and host cells, potentially leading to not only a unique combination of genetic and epigenetic aberrations but also a distinct microenvironment [2, 3]. Therefore, there exists an important need to identify predictive factors for response to immunotherapy beyond tumor MSI status and to develop effective combination treatment strategies.

Aspirin is a common nonsteroidal anti-inflammatory drug (NSAID) that inhibits *PTGS1* (cyclooxygenase-1) and *PTGS2* (cyclooxygenase-2), and regular aspirin use has been shown to reduce colorectal cancer incidence and mortality [Drew DA, et al. Nat Rev Cancer. 2016;16:173-186]. Taking the inter-tumor heterogeneity into account, large cohort studies suggest that survival benefits from postdiagnosis aspirin use are pronounced for colorectal cancer with *PTGS2* overexpression or activating *PIK3CA* mutations [4, 5] [Domingo E, et al. J Clin Oncol. 2013:31:4297-4305; Gray RT, et al. Clin Transl Gastroenterol. 2017:8:e91], supporting the potential of these molecular alterations as tumor biomarkers for response to adjuvant aspirin therapy. Notably, these findings provide population-based evidence for the underlying mechanisms by which aspirin may suppress colorectal cancer progression: i.e., aspirin may reduce colorectal cancer mortality by inhibiting *PTGS2* and prostaglandin E2 (PGE2) synthesis that are enhanced by activated PI3K signaling. Beyond these potential anti-tumor effects associated with inhibition of oncogenic signaling pathways, accumulating evidence points to immune-enhancing effects of aspirin on adaptive and innate immune response, including T cell-mediated anti-tumor immunity [6].

**Figure 1:**
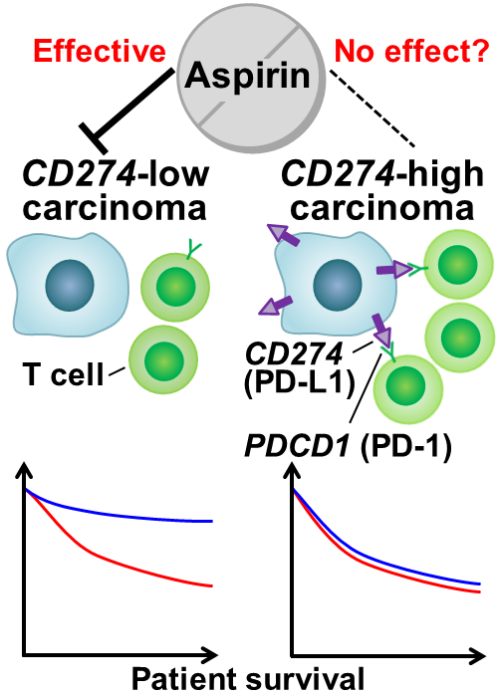
Postdiagnosis aspirin therapy and colorectal cancer progression according to tumor *CD274* (PD-L1) expression status

Recently, we reported a U.S. population-based study which suggested a stronger survival association of postdiagnosis aspirin use in colorectal cancer with lower-level *CD274* (PD-L1) expression than in cancer with higher-level *CD274* expression (Figure 1) [7]. This study was motivated by experimental data supporting a synergistic effect between aspirin and anti-*PDCD1* antibody [8]. In the study by Zelenay et al. [8], PTGS (cyclooxygenase)-induced PGE2 was shown to play a key role in suppressing anti-tumor immune response and promoting growth of cancer cells through increased levels of inflammatory mediators. In a mouse model, PTGS inhibition via aspirin synergized with the immune checkpoint blockade toward immune eradication of tumors. Based on these experimental and preclinical findings, we analyzed 617 colon and rectal carcinoma patients from two large prospective studies, and examined the differential survival association of postdiagnosis aspirin use by tumor *CD274* expression status, as evaluated by immunohistochemistry [7]. We found significantly lower colorectal cancer-specific mortality associated with postdiagnosis aspirin use in *CD274*-low tumors, but not in *CD274*-high tumors (*P*_interaction_ < 0.001). This differential survival association was consistently observed regardless of levels of tumor-infiltrating lymphocytes defined by histopathologic examination. These findings indicate that activation of the *CD274*-*PDCD1* immune checkpoint pathway may confer resistance to aspirin therapy, and that this could be overcome with the immune checkpoint blockade. This study was the first population-based study to suggest an interaction between immune checkpoint status and PGE2 inhibition via aspirin in regulating the progression of human colorectal cancer.

These aspirin studies were based on the paradigm of molecular pathological epidemiology (MPE) for survival analyses [2, 3]. In contrast to conventional epidemiology which examines the relationship of an exposure of interest with the outcome of patients grouped into a single disease entity (e.g., colorectal cancer), MPE research classifies the disease by means of tumor molecular and pathologic characterizations, and elucidates differences in treatment outcomes according to patterns of molecular alterations (e.g., *CD274*-low vs. *CD274*-high). Furthermore, the aspirin studies utilized the methodology of pharmacoepidemiology, clearly illustrating the successful integration of this traditional discipline with MPE (termed “pharmaco-MPE”) [3]. Importantly, these studies highlight the notable strengths of pharmaco-MPE: i.e., pharmaco-MPE research can 1) refine the effect estimate of the association of a medication with the outcome of a specific disease subtype, thereby identifying potential biomarkers for clinical benefits; 2) provide evidence on underlying mechanisms of benefits or harms of the medication; and 3) help develop tailored treatment strategies. As is clear from these features of MPE, MPE has emerged and evolved in parallel with the global trend toward precision medicine.

In summary, our study [7] underscores not only the potential role of the immune checkpoint status in modulating survival benefits of adjuvant aspirin therapy for colorectal cancer, but also the potential synergism of aspirin and the immune checkpoint blockade for combination immunotherapy strategies. These findings provide supporting evidence for future clinical studies investigating aspirin therapy in a target population (i.e., patients with *CD274*-low colorectal cancer) and in those receiving immune checkpoint inhibitors. Given the importance of better understanding of the tumor-immune microenvironment, consideration of immune parameters in MPE research would provide ample opportunities for development of novel immunomodulatory strategies of cancer prevention and treatment (which can be termed “immuno-MPE”) [3].
